# Submergence and Waterlogging Stress in Plants: A Review Highlighting Research Opportunities and Understudied Aspects

**DOI:** 10.3389/fpls.2019.00340

**Published:** 2019-03-22

**Authors:** Takeshi Fukao, Blanca Estela Barrera-Figueroa, Piyada Juntawong, Julián Mario Peña-Castro

**Affiliations:** ^1^School of Plant and Environmental Sciences, Virginia Tech, Blacksburg, VA, United States; ^2^Laboratorio de Biotecnología Vegetal, Instituto de Biotecnología, Universidad del Papaloapan, Tuxtepec, Mexico; ^3^Center for Advanced Studies in Tropical Natural Resources, National Research University – Department of Genetics, Faculty of Science, Kasetsart University, Bangkok, Thailand

**Keywords:** hypoxia, anoxia, biotechnology, cell signaling, stress perception, submergence, waterlogging

## Abstract

Soil flooding creates composite and complex stress in plants known as either submergence or waterlogging stress depending on the depth of the water table. In nature, these stresses are important factors dictating the species composition of the ecosystem. On agricultural land, they cause economic damage associated with long-term social consequences. The understanding of the plant molecular responses to these two stresses has benefited from research studying individual components of the stress, in particular low-oxygen stress. To a lesser extent, other associated stresses and plant responses have been incorporated into the molecular framework, such as ion and ROS signaling, pathogen susceptibility, and organ-specific expression and development. In this review, we aim to highlight known or suspected components of submergence/waterlogging stress that have not yet been thoroughly studied at the molecular level in this context, such as miRNA and retrotransposon expression, the influence of light/dark cycles, protein isoforms, root architecture, sugar sensing and signaling, post-stress molecular events, heavy-metal and salinity stress, and mRNA dynamics (splicing, sequestering, and ribosome loading). Finally, we explore biotechnological strategies that have applied this molecular knowledge to develop cultivars resistant to flooding or to offer alternative uses of flooding-prone soils, like bioethanol and biomass production.

## Introduction

The interaction of plants with the environment is a continuous process in which water plays a central role. At a global scale, indicators such as potential evapotranspiration and the number of wet days per year (amount and temporal occurrence of rainfall) directly determine the distribution of plants and species richness in a geographical context (Kreft and Jetz, 2007). In addition to rainfall, depth of the groundwater table, which is the interface between oxygenated soil and water-saturated aquifers, has a significant influence on maintaining aquatic ecosystems in dry periods, impeding drainage and defining wetland habitats for plants adapted to waterlogged soils (Fan et al., 2017). Together, rainfall and groundwater table depth influence soil hydrology, controlling physiological characteristics in plants to adapt to changes in water availability, such as rooting depth (Fan et al., 2017).

Agriculture around the world is constantly challenged by the rising incidence of adverse weather events as a consequence of global warming. Extreme events that alter water availability, like droughts and floods, constitute big threats to food security (Food and Agriculture Organization of the United Nations [FAO], 2017). Flooding of agricultural fields is generally caused by intensive and/or extensive rainfall over a period of time, but it may also result from overflowing of a body of water over land. Floods have a negative impact on economic and social aspects. Floods cause loss of livestock and seed stocks, destruction of infrastructure, machinery and tools, food shortage, diseases, and loss of agricultural productivity. This makes affected farmers and the general population vulnerable, causing poverty and migrations (Saldaña-Zorrilla, 2008).

On a global scale, floods were the cause of almost two-thirds of all damage and loss to crops in the period between 2006 and 2016, with a value of billions of dollars (Food and Agriculture Organization of the United Nations [FAO], 2017). For example, in Pakistan, extreme monsoon rains between 2010 and 2014 caused floods on large extensions of land that were responsible for the significant loss of at least 11 billion tons of rice, sugarcane, maize, and cotton, contributing to a total economic loss of over 16 billion dollars (Rehman et al., 2015). In addition, depending on the extent of the damage, resuming farming after a flooding event may require following an efficient recovery plan in order to remediate productive lands, which includes removing sediments, repairing physical and nutrient soil properties, reverting loss of beneficial soil microbial activity, and if the plant damage was lethal, replanting (Singh et al., 2013; Rey et al., 2019).

Based on the height of the water column produced, flooding can be classified as waterlogging, when it is superficial and covers only the root, or as submergence, when water completely covers the aerial plant tissues (Sasidharan et al., 2017). Both types of flooding disrupt the movement of oxygen from the air to plant tissues (Lee et al., 2011), producing a natural condition known as hypoxia (<21% O_2_) (Sasidharan et al., 2017).

At the agronomic level, there are different strategies to cope with submergence or waterlogging, such as developing standard models for the prediction and assessment of crop loss to floods for risk management, economic insurance, and decision making. Systems have been designed to predict flood-affected and flood-destroyed crop areas based on correlations to standard climate indices (Zhang Q. et al., 2016) or to estimate flooded crop acreage, crop damage and flood frequency and use algorithms to support post-flood crop loss estimation (Di et al., 2017). These systems constitute useful tools for planning, managing and applying solutions for the mitigation of damage to crops caused by floods.

In addition, the use of biological knowledge to breed superior cultivars can act as “genetic insurance” to guarantee a superior yield in the face of a flooding event, or even in multi-stress conditions (Mickelbart et al., 2015). In this sense, an excessive water supply induces hypoxia in plants (Lee et al., 2011), increases the vulnerability to pathogen attack (Hsu et al., 2013), and limits the flow of light to the plant (Jackson and Ram, 2003). During recovery after a flooding event, plants experience oxidative stress (Yeung et al., 2018) and must remobilize nutrients to achieve a normal homeostatic state (Tsai et al., 2016). Plants respond to flooding and the associated stress by changes in gene expression that are finely regulated at a multilevel scale from epigenetics (Tsuji et al., 2006) to transcriptional (Mustroph et al., 2010; Lee et al., 2011) and translational regulation (Juntawong et al., 2014a).

In recent years, flooding stress and its derivatives like submergence, waterlogging, hypoxia, and anoxia, were investigated extensively in plants, mainly in *Arabidopsis* and rice, to identify molecular elements that may play a role in tolerance to flooding. In this review, we intend to highlight aspects of this compound stress that present a niche of opportunity for expanding our knowledge and identifying genomic determinants in order to develop climate-resilient varieties that can meet the food demands of a rising world population.

## Advances on Stress Molecular Sensing and Signaling

Submergence and waterlogging trigger dramatic changes in gene expression, which coordinate morphological and metabolic adaptations to the stress (Bailey-Serres and Voesenek, 2008; Mustroph et al., 2010). Group VII ethylene response factors (ERF-VIIs) is a class of ERF transcription factors (TFs) that regulate the expression of a wide range of genes involved in adaptive responses to flooding and low oxygen (Voesenek and Bailey-Serres, 2015). In *Arabidopsis*, five ERF-VII genes, *HRE1*, *HRE2*, *RAP2.2*, *RAP2.3*, and *RAP2.12*, were recognized as key regulators for flooding and low-oxygen tolerance. These ERF-VIIs regulate a similar set of hypoxia-responsive genes, but constitutively expressed *RAP2.2*, *RAP2.3*, and *RAP2.12* are more powerful activators than stress-inducible *HRE1* and *HRE2* according to transactivation studies (Bui et al., 2015; Gasch et al., 2016). In rice, an ERF-VII TF gene, *SUB1A*, is known as the master regulator of submergence tolerance, allowing plants to endure complete submergence for 14–16 days (Fukao et al., 2006; Xu et al., 2006). Other rice ERF-VII genes, *SNORKEL1* and *SNORKEL2*, were found specifically in deepwater rice (Hattori et al., 2009). These tandemly repeated ERF-VIIs are responsible for enhanced internode elongation under flooded conditions, enabling plants to outgrow floodwaters. The importance of ERF-VIIs in flooding responses and tolerance is also indicated in *Rumex* and *Rorippa*, dicot species from flood-prone environments (van Veen et al., 2014).

The stability of most ERF-VII proteins is regulated by the N-end rule of proteolysis (NERP; Gibbs et al., 2011; Licausi et al., 2011a; van Dongen and Licausi, 2015). This sequential pathway has been recognized as a direct oxygen-sensing mechanism in plants because one of the enzymes in this process, plant cysteine oxidase (PCO), requires molecular oxygen as a co-substrate (White et al., 2017). The distinct expression patterns, ERF-VII selectivity, and catalytic capability of five *Arabidopsis* PCO isoforms suggest that they may have different biological roles in stress response and developmental regulation (White et al., 2018). Under ambient oxygen conditions, ERF-VII proteins are constitutively degraded via the N-end rule pathway. Under low oxygen, however, the oxygen-dependent PCO reaction is inhibited, resulting in the escape of ERF-VII proteins from targeted proteolysis. All *Arabidopsis* ERF-VIIs are substrates of the N-end rule pathway due to their conserved N-terminal MC motif (MCGGAI) that is recognized by the NERP pathway enzymes (Bailey-Serres et al., 2012; White et al., 2018). However, some rice ERF-VIIs such as SUB1A and SUB1C are not degraded via this pathway *in vitro* (Gibbs et al., 2011). In the case of SUB1A, the N-degron is protected from recognition through protein-protein interactions, and act during submergence through other downstream ERF-VIIs that are transcriptionally activated and susceptible to degradation by the NERP pathway (Lin et al., 2019). The stability of SUB1A protein in the presence of oxygen reflects enhanced recovery after de-submergence and increased tolerance to post-submergence injury in rice genotypes carrying *SUB1A* (Fukao et al., 2011; Alpuerto et al., 2016). SUB1C does not have an MC motif. Both *SNORKEL* ERFs have a minimum MC motif (Gibbs et al., 2011) but they have not been investigated as NERP substrates.

A recent study revealed that accumulation of the AtRAP2.12 protein (an *Arabidopsis* ERF-VII) is enhanced by a Raf-like mitogen-activated protein kinase kinase kinase (MAPKKK), HCR1, under combined oxygen-deprived and K^+^-sufficient conditions (Shahzad et al., 2016). Without exogenous application of K^+^, the effect of HCR1 on RAP2.12-mediated hypoxia responses, such as increased expression of core hypoxia-response genes (*CHG*) and reduced root hydraulic conductivity, was not observed even under oxygen deprivation. Under flooded conditions, the concentration of K^+^ in the soil is substantially lowered, and the uptake of K^+^ by plants is also restricted due to reduced hydraulic conductivity. Thus, this K^+^-dependent process under hypoxia suggests that low oxygen and flooding can increase internal K^+^ concentration in specific tissues and cell-types due to altered ion channel activities. Low oxygen regulates the function of various K^+^ channels in mammals (Wang F. et al., 2017). Further studies are required to elucidate the significance of K^+^ regulation in ERF-VII-mediated low-oxygen responses.

**FIGURE 1:**
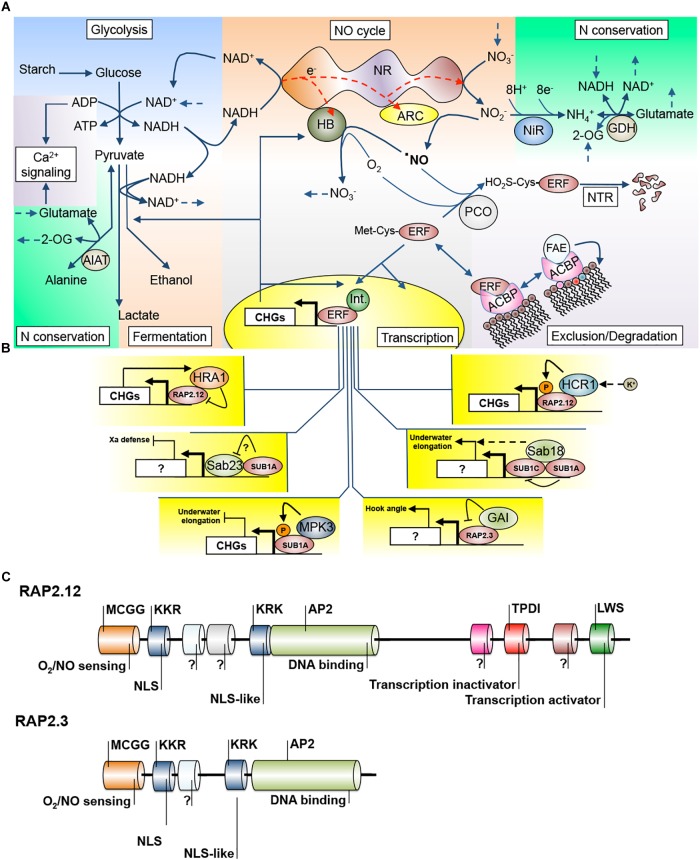
Interconnection of different signaling and transduction events during waterlogging/submergence stress. **(A)** Integration of metabolic pathways (glycolysis, fermentation, nitrogen conservation and assimilation), hormone homeostasis (NO cycle), calcium-mediated energy signaling (Ca^2+^ signaling), transcription factor (TF) abundance (N-terminal rule, NTR) and availability (membrane exclusion), and transcriptional control. **(B)** Known nuclear interactions of hypoxia/anoxia TFs with regulating proteins. **(C)** Comparison of the domain structure of RAP2.12, a high-complexity ERF-VII TF (the other being RAP2.2) and RAP2.3, a low-complexity ERF-VII TF (others being HRE1 and HRE2), and details of demonstrated domain functions. 2-OG, 2-oxoglutarate; ACBP, acyl-CoA binding protein; ALAT, alanine aminotransferase; ARC, amidoxime reducing component; CHGs, hypoxia core genes; ERF, ethylene response factor; FAE, fatty acid elongase; GAI, gibberellic acid insensitive; GDH, glutamate dehydrogenase; HB, hemoglobin; HCR1, hydraulic conductivity of the root; HRE1, hypoxia response attenuator; Int., interactor protein; MPK3, MAP kinase 3; NiR, nitrite reductase; NO, nitric oxide; NTR, N-terminal route; PCO, plant cell oxidase; SAB, Sub1A binding.

Another mechanism that controls the participation of ERF-VIIs in transcriptional activation is sequestration of the TFs to the plasma membrane (Licausi et al., 2011a). Under normoxia, RAP2.12 protein physically interacts with plasma membrane-localized acyl-CoA-binding proteins, ACBP1 and ACBP2, allowing RAP2.12 to escape from the N-end rule pathway (Figure 1A). In contrast, low oxygen promotes the removal of RAP2.12 from the plasma membrane, leading to relocalization of the TF to the nucleus (Kosmacz et al., 2015).

The transactivation capability of RAP2.12 is also regulated by a hypoxia-inducible trihelix TF, HRA1 (Giuntoli et al., 2014). HRA1 physically binds to RAP2.12, reducing the expression of CHGs. Interestingly, RAP2.12 stimulates mRNA accumulation of *HRA1*, indicating that HRA1 and RAP2.12 are regulated by a negative feedback loop. It was proposed that this regulatory mechanism can contribute to the avoidance of RAP2.12 overaccumulation, preventing rapid depletion of carbohydrate reserves under oxygen deprivation. Indeed, artificial stabilization of RAP2.12 by N-terminal modification (N-end rule insensitive mutation) reduced tolerance to low oxygen in *Arabidopsis* (Paul et al., 2016), emphasizing the significance of fine-tuning ERF-VIIs in hypoxia adaptation.

Many other protein–protein interactions of ERF-VIIs are described in the literature, opening a research area that may render much information (Figure 1B). SUB1A was reported to have dozens of protein partners in rice named Sub1A Binding Proteins (SAB; Seo et al., 2011). Only two of those interactions were characterized, with SAB23 and SAB18, and it was concluded that they improve the crosstalk between submergence stress and pathogen defense and the modulation of elongation, respectively (Seo et al., 2011). SUB1A also interacts in a positive feedback loop with mitogen-activated protein kinase 3 (MAPK), a central regulator of primary signaling cascades (mainly ROS), and allows submergence to suppress gibberellin action to inhibit elongation under submergence (Singh and Sinha, 2016). Fukao and Bailey-Serres (2008) demonstrated that SUB1A enhances inhibition of gibberellic acid (GA) signaling by the up-regulation of rice DELLA proteins, known negative regulators of GA. Recently, it was demonstrated that the *Arabidopsis* ERF-VII RAP2.3 physically interacts with DELLA proteins through a domain located on the N end of RAP2.3, regulating apical hook development (Marín-de la Rosa et al., 2014). When domains are analyzed in the structure of *Arabidopsis* ERF-VIIs, a division in low and high domain complexity can be observed (Figure 1C) (Nakano et al., 2006; Bui et al., 2015; Papdi et al., 2015). If we add that some of these ERFs are under transcriptional (Syed et al., 2015; Rivera-Contreras et al., 2016) and translational regulation by light (Loreti et al., 2018), we have a scenario of high versatility of potential protein–protein interactions providing the plant cell with ERF-VII regulation plasticity, but which has yet to be fully understood.

## Oxidative Stress and Energy Signaling and Management

### Stress/Post-stress Management of Oxidative Stress Symptoms

During flood events, plants are partially or completely submerged in water, but as floodwaters subside, plants suddenly encounter reoxygenation. Reoxygenation has been recognized as an abiotic stress that can injure plants post-submergence. For example, when rice plants were de-submerged from 7 days of inundation, an indicator of oxidative damage, malondialdehyde (MDA), along with superoxide anion and H_2_O_2_, was accumulated in the leaves (Fukao et al., 2011). Re-exposure to atmospheric oxygen after 7–10 days of submergence also induced leaf dehydration in rice (Setter et al., 2010; Fukao et al., 2011). These observations indicate that a plant’s survival of a flood event requires tolerance to multiple stresses such as submergence, reoxygenation, and dehydration. Indeed, the major submergence tolerance regulator in rice, *SUB1A*, confers tolerance to oxidative stress and dehydration through activation of ROS detoxification and abscisic acid (ABA) responsiveness (Fukao et al., 2011). Similarly, in *Arabidopsis* many ERF-VIIs are involved in the adaptation to submergence, oxidative stress, and dehydration (Tamang and Fukao, 2015). A recent study demonstrated that jasmonate is a pivotal hormone to activate ROS detoxification systems under reoxygenation in *Arabidopsis* (Yuan et al., 2017). Exogenous application of jasmonate increased tolerance to reoxygenation, whereas jasmonate-deficient mutants exhibited intolerance to this stress.

### Nitric Oxide as a Central Homeostatic Regulator of Hypoxic Stresses

The accumulated knowledge on the biological roles of nitric oxide (NO) in plants fits well with a modern model where ROS are increasingly recognized as an integral part of the cellular signaling process (Foyer et al., 2017). This view is reinforced when taking into account the environment in which actual flooding/submergence stresses occur. In the field, plants still must balance the presence and absence of varying degrees of illumination. That rarely will create a complete shutdown of aerobic and anabolic pathways in favor of fully anaerobic and catabolic pathways, but rather a complex situation where balance is intended. In addition, the stress will not arrive as a sudden insult; it will build up gradually around the plant.

Recent research in the unicellular alga *Chlamydomonas reinhardtii* highlighted the enzyme nitrate reductase (NR) as a versatile platform to achieve this balance. Using genetic and pharmacological tools, it was demonstrated that NR provides the structure for a short, cytosolic electron transport chain that is able to distribute electrons, not only through its canonical pathway from NAD(P)H to nitrite, but also to NO through the interacting protein amidoxime reducing component (ARC), and to NO and O_2_ through hemoglobin (HB) to form a cycle back to nitrate (Chamizo-Ampudia et al., 2016) (Figure 1A). These authors proposed a hypothetical structural model that would spatially accommodate all these alternative electron transfers in a dimeric NR association (Chamizo-Ampudia et al., 2017).

Interestingly, blocking NO production through the genetic knockout of NR genes is sufficient to stabilize ERF-VII proteins even under normoxic conditions, indicating that NR is the main source of NO in plant cells (Gibbs et al., 2014). Therefore, being a cytoplasmic complex interacting with soluble and diffusible electron carriers (Chamizo-Ampudia et al., 2016) with gene homologs in both dicot and monocot model plants (Tejada-Jiménez et al., 2013), NR/ARC/HB would be able to connect most of the known hypoxia/anoxia responses such as glycolysis, N assimilation, NO signaling, and ERF-VII activity (Figure 1A). The finding that *Arabidopsis* ARC is able to catalyze the *in vitro* production of NO (Yang et al., 2015) opens the possibility of further exploration of the biochemistry and applications of the NR/ARC/HB electron chain transport.

Acting as a meeting hub of survival strategies, NR/ARC/HB has several levels of plasticity. Transcriptionally, both in *Arabidopsis* and *Brachypodium*, *NR* transcripts are down-regulated in shoots by submergence, *HB* transcripts are strongly up-regulated and are an integral part of the CHGs, and *ARC* transcripts (At1g30910, At5g44720, Bradi1g32350, and Bradi4g37990) are mildly down-regulated (Lee et al., 2011; Rivera-Contreras et al., 2016). However, in tomato and tobacco, despite the down-regulation of *NR* mRNA under root hypoxia, the activity of NR is increased to a level where external nitrate protects against stress damage even in aerial parts (Allègre et al., 2004). On the other hand, excessively low NR activity in tobacco roots leads to high lactic acid and ethanolic fermentation, but without the protective effect of nitrite against H^+^ cytoplasmic acidification (Stoimenova et al., 2003; Libourel et al., 2006). At the protein level, these observations indicate a stress scenario where the core NR structure production is limited, its activity maintained, and its two alternative electron acceptors, ARC and HB (paradoxically working in sequence), remain to compete with a strong balance toward NO removal and the expected consequence of homeostatic regulation of anaerobic metabolism (Figure 1A). In *Jatropha curcas* this logic is not followed since NR transcripts are up-regulated and this was considered a probable cause of waterlogging sensitivity (Juntawong et al., 2014b).

The abundance of NR protein under normoxia is positively regulated by nitrate and negatively by ammonia (Kim et al., 2018). These observations concur with measurements in hypoxic roots where nitrate is actively reduced to nitrite and NO, but this activity is inhibited by external ammonia, and as a consequence fermentative lactate production increases (Oliveira et al., 2013). Studies have shown that, during hypoxia, both nitrate and ammonia tend to increase; ammonia improves plant fitness, indicating the need to balance the canonical NR activity to support nitrogen fixation (Allègre et al., 2004).

The stability of NR protein in normoxia is also under different post-translational controls with positive effects like sumoylation, or negative ones like phosphorylation and proteasome degradation (Park et al., 2011), and intriguingly, by partitioning its subcellular location to the nucleus (Kim et al., 2018). Interestingly, it was found that NR is destabilized by CONSTITUTIVE PHOTOMORPHOGENIC 1 (COP1), a ubiquitin ligase (Park et al., 2011). In *Rumex palustris*, a plant that can tolerate submergence, higher *COP1* and *HB* expression were measured concomitant with lower ammonia and higher nitrate and protein concentrations (even in normoxia) when compared to *Rumex acetosa*, which is sensitive to submergence, thus indicating that posttranslational suppression of NR may be a relevant tolerance mechanism (van Veen et al., 2013). Therefore, NR/ARC/HB provides an interesting genetic platform to develop biotechnological strategies for improving plant performance under stress (Llamas et al., 2017).

The biological relevance of the NO cycle (Igamberdiev and Hill, 2004) to fix NO by NR and channel it to nitrogen assimilation pathways was tested *in vivo*. Under nitrogen-limiting conditions it was shown that externally applied NO can overcome N deficiency and was improved by HB overexpression (Kuruthukulangarakoola et al., 2017). Therefore, under hypoxia, NO recycling can simultaneously provide NAD^+^ for glycolysis and to allow alanine synthesis to compete with the fermentative pathways and conserve nitrogen (Figure 1A). An important nitrogen recycling pathway seems to be that of glutamate dehydrogenase (GDH), because under anoxic stress the *Arabidopsis gdh* double mutant accumulates more 2-oxoglutarate, has lower gamma-aminobutyric acid (GABA) reserves, and is deficient in alanine consumption during reoxygenation (Tsai et al., 2016). These metabolic defects have dramatic negative consequences for plant survival after submergence.

Although GDH is known to operate *in vivo* in the deamination of glutamate (Labboun et al., 2009), a role for amination activity under oxygen stress is suggested not only by the accumulation of 2-oxoglutarate in the *gdh* mutant but also because ^15^NH_4_ is barely incorporated into glutamine in soybean roots (implying that ATP limits glutamine synthase, the regular ammonium assimilation pathway) but still is efficiently transferred to GABA and alanine (António et al., 2016). Since plants have covered their needs to balance carbon and nitrogen with at least four major enzymes (alanine aminotransferase, aspartate amino transferase, GABA transaminase and GDH), untangling this metabolic network requires experimental models that take into account the nitrogen source, the use of external sucrose in the analysis buffers, methodological invasiveness, and microenvironment control. Recently, an elegant multidisciplinary approach was used to demonstrate the role of GDH in recycling ammonia to sustain the proliferation of cancer cells (Spinelli et al., 2017).

### Energy and Calcium Signaling

In *Arabidopsis*, the translocation of ERF-VII protein RAP2.12 to the nucleus is dependent on the oxygen concentration (Kosmacz et al., 2015) and the dioxygenase enzymatic activity of PCO isoforms reaches its peak at 20% oxygen, further declining almost linearly as O_2_ decreases; the precise relationship between NO and O_2_ *in vivo* is still unknown, however (White et al., 2018). This recent knowledge indicates that plant cells have evolved efficient mechanisms to sense oxygen levels and to transduce those concentrations into a molecular response suited to face different flooding and hypoxic scenarios.

Recent reviews have proposed that this oxygen-sensing capacity must be accompanied by other sensing and transduction mechanisms, especially aimed at detecting a cellular energy crisis. By comparison to animal cells, several candidate mechanisms were proposed to react at a faster rate than the oxygen sensing capability of ERF-VII, and most of them rely on membrane proteins acting as ionic transporters (Wang F. et al., 2017). Igamberdiev and Hill (2018) have presented convincing arguments for the role of calcium signaling as a strong, fast and flexible primary signal of energy stress, in particular the increase in the level of cytoplasmic calcium [Ca^2+^]_cyt_ from internal and apoplastic sources, but more importantly, directly from altered ATP/ADP ratios.

Temporary spikes of [Ca^2+^]_cyt_ and subsequent cellular efforts to return to a homeostatic level were measured in anoxic and recovery conditions more than 20 years ago (Sedbrook et al., 1996). Later, it was demonstrated that an H_2_O_2_-mediated cellular rheostat composed of Rop G-proteins and its interactor RopGAP involves [Ca^2+^]_cyt_-activated NADPH oxidases and that a misbalance of any component leads to defects in alcohol dehydrogenase (ADH) expression with large impacts on plant survival after hypoxic stress (Baxter-Burrell et al., 2002). Calcium can also regulate ATP-Mg/Pi mitochondrial transporters, providing a direct pathway to flexibly regulate the ATP/ADP pool (Stael et al., 2011).

Successful return to a homeostatic state of [Ca^2+^]_cyt_ is also important for plant fitness, since knockout mutants for Ca^2+^ transporters of ATPases and proton antiporter families (ACA/CAX) present aberrant concentrations of Na^+^, K^+^, and Ca^2+^. Remarkably, this inability to correct [Ca^2+^]_cyt_ leads to lower biomass after waterlogging stress (Wang et al., 2016). The importance of crosstalk between [Ca^2+^]_cyt_-sensitive systems and H_2_O_2_ modulation was demonstrated by the characterization of the CHG coding for HRU1, a protein belonging to the archaic universal stress protein (USP) family, which is capable of physically interacting with the RopGAP rheostat and whose misregulation leads to severely sensitive phenotypes under submergence and anoxic stresses (Gonzali et al., 2015). Remarkably, the expression of *Arabidopsis HRU1* is dependent on ERF-VII, allowing it to get feedback from oxygen sensing. In tomato, another member of the USP family is able to interact with a Ca^2+^-dependent protein kinase to physically transduce [Ca^2+^]_cyt_ to ROS signaling (Gutiérrez-Beltrán et al., 2017).

Finally, downstream transducers of [Ca^2+^]_cyt_, like calmodulin-like protein, connect this elemental signal to more complicated processes such as the formation of multimolecular ribonucleoprotein complexes that, when disrupted, have large effects on plant survival of hypoxia (Lokdarshi et al., 2016) (see section “RNA Dynamics Under Stress”), most likely by the incorporation of efforts by plant cells to save energy through the conservation of mRNAs that are useful during the expected reoxygenation (Sorenson and Bailey-Serres, 2014). These complexes have multiple connections to other stresses and cellular homeostatic processes (Chantarachot and Bailey-Serres, 2018).

With all this research it is becoming more clear that this multilevel signaling and transduction network is in agreement with the evidence that anaerobic responses are not unique to hypoxic stress and provide a unifying genetic framework to generically respond to stress conditions that decrease energy availability (Greenway and Armstrong, 2018) and, at the same time, provide mechanisms to sense O_2_ concentrations (Sasidharan et al., 2018), thus creating an adjustable response to the evolutionary range of stress intensities found in nature by combining in time energy stress, waterlogging and submergence peculiarities and light/dark cycles.

Recently, by means of high-definition live imaging, genetically encoded fluorescent sensors and genetics, observations on the scale of seconds could be made of the fast dynamics of [Ca^2+^]_cyt_ bursts, signal distribution, and homeostatically set points in intact plants in the context of wounding/herbivore stress and how it is mediated, almost exclusively, by glutamate and glutamate transporters (Toyota et al., 2018). This amino acid is a pivotal metabolite of hypoxic stresses (Barding et al., 2013). This imaging research strategy should shed light on the dynamic connections of the many signaling hubs reviewed here, from both the biological and biotechnological aspects of flooding stress.

## Molecular Mechanisms of Multicomponent Stress

### Root Architecture and Responses

Root architecture and plasticity play an important role in the adaptation to submergence and waterlogging stress. The formation of aerenchyma and adventitious roots is a morphological characteristic of waterlogging-tolerant species. Aerenchyma is known to enhance internal oxygen diffusion from the aerial parts to the waterlogged roots that allows the roots to maintain aerobic respiration (Armstrong, 1980).

Two types of aerenchyma can be found in roots: primary aerenchyma, consisting of lysigenous aerenchyma, and schizogenous aerenchyma in the roots of rice, maize and wheat, and secondary aerenchyma in soybean roots (reviewed in Yamauchi et al., 2018). The formation of lysigenous aerenchyma results from the selective death and subsequent lysis of root cortical cells, while schizogenous aerenchyma formation is caused by cell separation, without the occurrence of cell death. Secondary aerenchyma develops from phellogen, forming spongy tissue filled with air spaces outside of the stem, hypocotyl, and roots.

Gene expression analysis was applied to study the molecular mechanisms of lysigenous aerenchyma formation in roots using a microarray combined with laser microdissection demonstrating that the expression of genes involved in calcium signaling, cell-wall modification, ethylene and reactive oxygen species (ROS) changes during lysigenous aerenchyma formation under conditions of low oxygen (Rajhi et al., 2011; Yamauchi et al., 2011). However, the precise molecular mechanism controlling both primary and secondary aerenchyma formation has not yet been characterized.

In addition, the spatial arrangement of roots and their components is crucial for the adaptation to submergence and waterlogging stress. In *Arabidopsis*, low-oxygen-induced primary root bending is controlled by hypoxia-induced auxin flux at the root tips but negatively regulated by the hypoxia-induced group-VII ERFs (Eysholdt-Derzsó and Sauter, 2017). Moreover, the hypoxia-induced group-VII ERFs also promote adventitious root (AR) elongation, while ethylene inhibits adventitious root formation (Eysholdt-Derzsó and Sauter, 2018). In *Solanum dulcamara*, a dicot species that constitutively develops dormant primordia that can be reactivated upon flooding, transcriptome analysis and hormonal treatment were used to investigate the signaling pathway controlling AR primordium reactivation (Dawood et al., 2016). Flooding increased ethylene accumulation and subsequently a drop in ABA level in AR primordia tissue. ABA treatment inhibited activation of AR primordia by flooding and blocking of ABA was able to reactivate AR primordia in the absence of flooding (Dawood et al., 2016). Both flooding and ethylene induced polar auxin transport, but auxin treatment alone was not sufficient for AR emergence indicating the interplay among ABA, auxin, and ethylene contributing to the regulation of AR development under flooding. In waterlogging-tolerant legumes of the genus *Trifolium*, its ability to tolerate waterlogging coordinates with higher root porosity and the ability to form lateral roots (Gibberd et al., 2001). However, little is known about the molecular mechanism in waterlogging-tolerant crops controlling lateral root formation induced by waterlogging.

Plants are able to respond to different levels of flooding and systemic signals can travel between underground and aerial organs, causing differential transcriptomic responses (Hsu et al., 2011); ethylene is one of these signals affecting many processes, including the characteristic hyponastic response in the leaves of many plants (Polko et al., 2015). However, many other signals can also come into play, for example, carbohydrates, ABA, ions, and amino acids, even ratios of different closely related metabolites, notably T6P and sucrose whose enzymatic control (through trehalose phosphate phosphatase) is expressed in young heterotrophic tissues, especially in the roots (Kretzschmar et al., 2015).

Therefore, understanding root architecture, development and plasticity under submergence and waterlogging stress could further clarify plants’ responses to the stress. Supporting this idea, Mustroph (2018) recently reviewed the vast literature of reported quantitative trait loci (QTL) associated with flooding stress and found that, remarkably, most QTLs associated with waterlogging resistance in economically relevant tolerant cultivars of grasses were related to aerenchyma and adventitious root formation, and require further molecular understanding.

### Carbohydrate Depletion Sensing

Under flooded conditions, plants experience energy and carbohydrate deprivation due to reduced photosynthesis and aerobic respiration. Plants encounter a similar energy status when exposed to prolonged darkness. Therefore, it is not surprising that plants tolerant to submergence can also endure prolonged darkness. Constitutive and stress-induced expression of *SUB1A* increases tolerance to submergence in rice through the restriction of carbohydrate breakdown and elongation growth (Fukao et al., 2006; Fukao and Bailey-Serres, 2008). The same genotypes exhibit stronger viability under prolonged darkness by reduced degradation of carbohydrate reserves and chlorophyll (Fukao et al., 2012). Under both submergence and constant darkness, *SUB1A* restricts the biosynthesis of ethylene, a hormone promoting leaf senescence. Similarly, mutation of *prt6* and its allelic variant, *ged-1*, increased both submergence and dark tolerance in *Arabidopsis* through the reduced breakdown of starch reserves (Riber et al., 2015). These results indicate that common signaling and metabolic pathways are involved in the regulation of submergence and dark tolerance in plants. Comparative microarray analysis revealed that 29.2% of genes up-regulated upon submergence in shoots are also induced by prolonged darkness in *Arabidopsis* (Lee et al., 2011), suggesting the existence of common regulatory pathways for submergence and dark tolerance.

Sucrose non-fermenting-1-related kinase 1 (SnRK1) is the central regulator of energy homeostasis in plants where its kinase activity is induced in response to energy starvation (Emanuelle et al., 2016). Besides the energy status, SnRK1 activity is also regulated by the redox status (Wurzinger et al., 2017). In rice, SnRK1A plays a critical role in seed germination under anaerobic (flooded) conditions (Lee et al., 2009). Submergence-induced sugar starvation triggers mRNA accumulation of calcineurin B-like protein-interacting protein kinase 15 (*CIPK15*), enhancing the accumulation of SnRK1A proteins. CIPK15 also physically interacts with SnRK1A, resulting in MYBS1-mediated alpha-amylase induction (Lee et al., 2009). This SnRK1A-mediated process is also pivotal for rice seed germination under non-stress conditions (Lu et al., 2007) because localized sugar starvation occurs in actively growing tissues even under aerobic conditions. Altering SnRK1 signaling in *Arabidopsis* had strong repercussions on plant tolerance, disrupting AMP/ATP ratios, T6P and amino acid homeostasis (Cho et al., 2016). Not surprisingly, starch is a crucial nutrient reservoir for tolerance to submergence; both inabilities to use and synthesize starch are detrimental for plant survival (Loreti et al., 2018). However, signaling of sugar availability works through gene redundancy of SnRK and the expression of basic leucine zipper TFs (bZIP TFs) in a separate pathway from the transcriptional regulation of CHGs (Loreti et al., 2018).

The role of SnRK1 in the regulation of carbohydrate and nitrogen metabolism under starvation conditions has been characterized. For example, SnRK1 directly phosphorylates HMG-CoA reductase, NR, sucrose phosphate synthase, 6-phosphofructo-2-kinase/fructose-2,6-bisphosphatase, and trehalose-6-phosphate synthase to regulate their enzymatic activities (Mackintosh et al., 1992; Douglas et al., 1997; Sugden et al., 1999; Kulma et al., 2004; Harthill et al., 2006). SnRK1 also activates S_1_-bZIPs such as bZIP1, bZIP11, and bZIP53, which regulate starch and branched-chain amino acid catabolism, trehalose-6-phosphate biosynthesis, and nitrogen signaling in *Arabidopsis* under starved conditions (Baena-González et al., 2007; Dietrich et al., 2011; Ma et al., 2011; Para et al., 2014). More recently, SnRK1 was reported to phosphorylate C-type bZIPs and promote the formation of C/S_1_ bZIP heterodimers (Mair et al., 2015; Pedrotti et al., 2018). The C/S_1_ bZIP–SnRK1 complex binds to target promoters and regulates chromatin structure via histone acetyltransferases (Pedrotti et al., 2018). The downstream genes directly or indirectly regulated by the SnRK1–bZIP network are associated with crucial metabolic pathways for plant survival under energy starvation.

A similar mechanism mediated by SnRK1 may regulate the expression of hypoxia-responsive genes. Indeed, SnRK1 directly binds to the promoter regions of hypoxia-inducible genes in response to submergence, which correlates with the increased expression of these genes in *Arabidopsis* (Cho et al., 2012). Because SnRK1 contains no DNA-binding domains, the authors suggested that protein complexes consisting of SnRK1 and TFs (e.g., C/S_1_ bZIPs) bind to the target promoters and modify their chromatin structure. Identification of TFs interacting with SnRK1 and target gene chromatins will advance the molecular understanding of SnRK1-mediated transcriptional regulation under submergence and waterlogging.

### Bacterial Damage and Preemptive Transcriptomic Preparation

Consistently, transcriptomic studies under submergence stress have found up-regulated gene clusters associated with plant responses to fungi and bacteria. Expressed genes code for pattern recognition receptor proteins (PRRs), wall-associated kinases (WAKs), leucine-rich repeats (LRRs) and lectin-DUF26 proteins (Lee et al., 2011; Hsu et al., 2013; Rivera-Contreras et al., 2016). The number of genes inside these clusters, ranging up to hundreds, suggests that complex plant–microbe relationships are expected by the plant cell to be counteracted/established concomitant to flooding. These can be detrimental, for example, an increased number of microbial cells with potential pathogenic capacities. On the other hand, the induction and stabilization of beneficial partnerships may also be possible.

Both of these possibilities were explored experimentally. Hsu et al. (2013) demonstrated in *Arabidopsis* that strong up-regulation of innate immunity genes can be detected as early as 1 h after submergence. Interestingly, a 2-h submergence stress treatment conferred the plant tolerance to post-submergence inoculation with *Pseudomonas syringae*. When T-DNA knockout mutants of TF *WRKY22* were simultaneously confronted with submergence and the bacterial pathogen, mutant plants suffered more visual damage and harbored a bacterial population of twice the size of that on wild-type plants. WRKY TFs are transcriptional modulators of plant–microbe interactions and have as genetic targets different LRRs, PRRs, and WAK proteins. Parallel to this pathway, Vicente et al. (2018) used mutations in PRT6, a pivotal N-terminal rule enzyme, and also observed increased resistance to *P. syringae* (in *Arabidopsis*) and to *P. japonica* and *Blumeria graminis* (in barley), most likely by stabilization of ERF-VII TFs (Vicente et al., 2018). Therefore, during submergence/waterlogging, WRKYs and ERF-VII TFs create a positive pathogen defense genetic loop.

The hundreds of pathogen-related genes expressed may be a reflection of an evolutionary history of exposition to probable pathogens, since some of these genes (e.g., lectins) were demonstrated to specifically recognize chemical patterns of certain species (Miyakawa et al., 2014). On the other hand, positive plant–bacteria relationships have also been documented during a flooding event. In rice, the diversity of nitrogen-fixing bacteria was studied before and after intentional flooding as part of normal agricultural practices (Ferrando and Fernández Scavino, 2015). The results indicate that flooding helped to establish a more diverse facultative/anaerobic diazotrophic community, especially in the rhizosphere. Breidenbach and Conrad (2014) also reported that planted rice soils have a stable population throughout plant development which changes in response to draining and crop rotation to maize. Although not directly related to flooding, a report in *Arabidopsis* (Lundberg et al., 2012) demonstrated that bacterial populations in the rhizosphere are distinct from those detected in soil and, more importantly, are characteristic of different genotypes, predicting that the signaling for bacterial recruitment in roots is genetically coded.

The works mentioned above successfully used available mass-sequencing technologies to overcome the methodological barriers imposed by traditional microbiology to study the complex rhizosphere environment. Although, the study in rice may represent the case of a domesticated plant adapted for flooding events where positive interactions develop, the experimental working frame employed by the authors may shed light on other crops and wild plants that may not be readily prepared to establish positive interactions in a flooding event and where defense triggering mechanisms may be more important.

Another unexplored pathway of plant–microbe interactions in submergence is the input of jasmonate-zim-domain protein 3 (JAZ3) to submergence survival given that it is a CHG in *Arabidopsis* (Gasch et al., 2016), a family member of first-transducers of [Ca^2+^]_cyt_ (Toyota et al., 2018) with important roles as both pathogen defense mediator and as a natural target for chemically induced susceptibility by fungal pathogens mimicking jasmonic acid signaling (Chini et al., 2018).

### Heavy Metals and Salinity

Transcriptomic studies of submergence stress have frequently reported the altered expression of transcripts coding for proteins of heavy-metal homeostasis (Lee et al., 2011; Rivera-Contreras et al., 2016). Also, a common pattern of ethylene and ROS bursts and responses was observed in heavy-metal stress and flooding stress signaling, pointing to probable co-evolution (Steffens, 2014). Measurements of ion concentrations during a flooding event indicate that Na, Al, B, Mn, and Fe tend to accumulate (Setter et al., 2009; Zeng et al., 2013) and, interestingly, that plants are able to restrain traffic of toxic ions to their biomass (Kashem and Singh, 2001). The ability to restrain this traffic is also differential and characteristic for different genotypes, as demonstrated in wheat (Setter et al., 2009).

The relevance of these observations to submergence/flooding tolerance is highlighted by the finding that transcripts coding for proteins of iron homeostasis are more strongly down-regulated in *Arabidopsis* roots of tolerant ecotypes (van Veen et al., 2016). Using RNA-Seq, Du et al. (2017) analyzed the response to waterlogging in roots of tropical maize lines and found a mRNA coding for a heavy-metal transporter as differentially expressed in tolerant lines. The genomic location of this gene coincided with a previous QTL associated with more root biomass under waterlogging in maize seedlings (Osman et al., 2013). This particular gene has not been molecularly characterized; therefore, its promising role under stress is poorly understood.

## RNA Dynamics Under Stress

### Cytoplasmic RNA Dynamics

Regulation of gene expression is an important factor controlling most plant biological processes, including cellular differentiation, organ development, and environmental adaptation to the constantly changing environment. In plants, the fundamental role of transcriptional regulation is universally recognized, and the general mechanism participating in transcriptional regulation is well understood. In recent years, post-transcriptional control has been embraced as an essential process controlling plant gene expression (Chantarachot and Bailey-Serres, 2018).

Submergence and waterlogging stress typically limit oxygen availability and energy production in plant cells. Several pieces of evidence demonstrate that post-transcriptional gene regulation participates in the metabolic adjustments that aid a plant’s surviving conditions of low oxygen. Cellular mRNAs typically associate with RNA-binding protein (RBP) forming mRNA–ribonucleoprotein (mRNP) complexes. These mRNP complexes determine the activity of individual RNAs through the regulation of mRNA processing, localization, translation, sequestration, and degradation (Bailey-Serres et al., 2009).

Plant cytoplasmic ribosome complexes are among the best characterized mRNP complexes. The ribosome complexes contribute to the initiation, elongation, and termination of translation. During initiation, the large subunit (60S) of the ribosome joins the small subunit (40S) at the AUG initiation codon, forming an 80S ribosome. The sequential recruitment of ribosomes onto a single mRNA generates polyribosome (polysome) complexes (Juntawong et al., 2014a). Using *Arabidopsis* expressing a FLAG-epitope tagged ribosomal protein L18, translating ribosome affinity purification (TRAP) technology can be used to isolate the mRNA populations associated with at least one 80S ribosome that could be further analyzed by high-throughput genomic approaches (Reynoso et al., 2015). TRAP allows quantitative examination of the plant translatome for changes in mRNA translation. A study using TRAP and microarrays demonstrated that a condition of low oxygen reduces the cellular ATP content and results in selective translation and global translational repression, which is rapidly reversible following reoxygenation in *Arabidopsis* (Branco-Price et al., 2008). Interestingly, a large proportion of mRNAs that display no changes in abundance and some low-oxygen-induced mRNAs are not translated until reoxygenation, suggesting these mRNAs could be sequestrated in non-translated mRNP complexes. The contribution of selective mRNA translation to low oxygen was further assessed in specific cell populations of *Arabidopsis* seedlings using the cell-type-specific TRAP technology combined with microarrays (Mustroph et al., 2009). The cell-type-specific translatome exposed a common general response in most cells but with complex and yet unexplored local molecular responses and adaptation to low oxygen.

Because a single mRNA can associate with more than one ribosome, ribosome profiling (Ribo-Seq) technology that includes the nuclease digestion of mRNAs associated with ribosomes, deep-sequencing of ribosome-protected footprints (RPFs) and mapping of RPFs to the transcripts, allows the quantification of translation rates of individual mRNAs (Juntawong et al., 2015). Evaluation of mRNA translation using Ribo-Seq in *Arabidopsis* seedlings subjected to low oxygen revealed several aspects of translational regulation (Juntawong et al., 2014a). Firstly, low oxygen globally reduces initiation but increases termination. Secondly, the translational efficiency of individual mRNAs is selectively controlled under conditions of low oxygen. Thirdly, translation of alternatively spliced mRNAs and non-coding (nc) RNAs could contribute to the proteomic diversity in response to low-oxygen conditions. Lastly, features such as an upstream Open Reading Frame (uORF) on individual mRNAs affect mRNA translation under conditions of low oxygen, including bZIP TFs, are pivotal to sugar signal transduction under different scenarios and are regarded as paradigms of uORF translation regulation (Dröge-Laser et al., 2018).

mRNA sequestration is considered a mechanism that contributes to the control of mRNA translation under low oxygen. In mammalian cells, energy and nutrient stresses result in global repression of translation followed by the recruitment of non-translationally active mRNAs into the stress granule (SG) mRNP complexes (Chantarachot and Bailey-Serres, 2018). SGs are typically recognized as mRNA storage sites that are involved in the dynamic exchange of mRNAs with translating ribosomes and processing bodies (PB) where the mRNAs are degraded (reviewed in Bailey-Serres et al., 2009). In plants, oligouridylate binding protein 1C (UBP1C), a triple RNA Recognition Motif (RRM) ortholog of the animal SG nucleated protein T-cell intracellular antigen 1 (TIA-1), is dynamically and reversibly associated with translationally inactive mRNAs forming UBP1C-SGs under conditions of low oxygen (Sorenson and Bailey-Serres, 2014). In addition, UBP1C knockdown lines display a low-oxygen-sensitive phenotype, indicating the significance of SG formation and mRNA sequestration as a mechanism controlling the response to low oxygen in plants.

Evidence of the functional roles of RNA dynamics in fine-tuning low-oxygen gene expression and the molecular mechanisms underlying these modes of regulation has been emerging gradually. Recent advances in high-throughput approaches that allow the identification and quantification of ribosome footprints, RNA structure, and protein–RNA interaction will facilitate the understanding of the mechanism controlling RNA dynamics toward low-oxygen response.

### Expression and Modulation of miRNAs

MicroRNAs (miRNAs) are endogenous, small non-coding RNAs that act as post-transcriptional regulators of gene expression and have been extensively described as being involved in biological processes in plants such as development and the response to environmental stimuli (Chen, 2009). In plants, miRNAs recognize target mRNAs by almost perfect base pairing and direct them for RNA cleavage or inhibition of translation (Rogers and Chen, 2013). When a specific miRNA is accumulated, the expression of the target mRNA is expected to be decreased, and vice versa. MiRNA expression is highly sensitive to environmental clues that prepare them to function as dynamic regulators of response to stress in a fine-tuned manner. Moreover, miRNAs act as regulatory nodes in complex networks to interconnect the response to biotic and abiotic stress with plant development (Rubio-Somoza and Weigel, 2011). With the advent of NGS technologies, several miRNAs were described, uncovering a diversity of biological functions in response to stress in plants.

**FIGURE 2:**
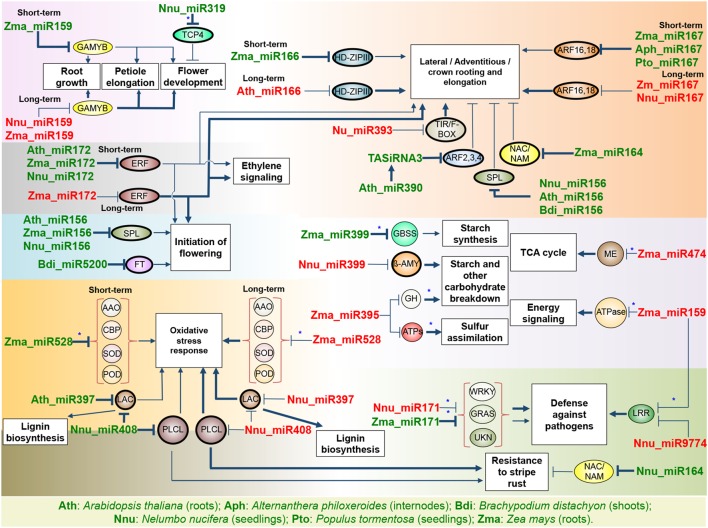
miRNA modulation of known responses to flooding. The name of a miRNA when green or red indicates up- or down-regulation under flooding stress, respectively. The thickness of the arrows indicates the enhancement of the activation/repression activity. Ovals indicate transcription factors while circles indicate enzymes; thick-lined circles indicate targets confirmed in the plant studied. When known, the short-term and long-term expression under flooding stress is indicated. Asterisk indicates a predicted unconfirmed target. AAO, ascorbic acid oxidase; ATPs, ATP sulfurylase; CBP, copper binding protein; GBSS, granule-bound starch synthase; GH, glycosyl hydrolase; LAC, laccase; LRR, leucine-rich repeat; ME, malic enzyme; PLCL, plantacyanin-like protein; POD, peroxidase; SOD, superoxide dismutase; UKN, unknown function protein; β-amy, beta-amylase.

miRNAs have an active role in response to flooding in plants as revealed by genome-wide studies in response to hypoxia (Moldovan et al., 2010; Licausi et al., 2011b), waterlogging (Liu et al., 2012; Zhai et al., 2013), and submergence (Zhang et al., 2008; Jeong et al., 2013; Jin et al., 2017; Li et al., 2017; Franke et al., 2018). Despite the fact that reports of miRNA’s responsiveness to flooding stress are still scarce in comparison to reports on other abiotic stress, existing studies have revealed that miRNAs regulate four main lines of response to flooding stress including morphological adaptation, management of energy supply, control of flowering, and oxidative stress response (Figure 2).

#### Morphological Adaptation

miRNAs promote and control the balance between the response to flooding stress and development through hormonal signaling pathways. The analysis of *cis*-regulatory elements in the promoters of flooding-responsive miRNAs has made evident that ethylene, GA, ABA, and auxin signal transduction pathways are main components interconnecting the complex network of response to flooding (Zhang et al., 2008; Liu et al., 2012). Downstream, several flooding-responsive miRNAs regulate target mRNAs, encoding TFs involved in hormonal signaling pathways that orchestrate adaptation to flooding through morphological changes in the different plant tissues.

For example, in the roots of maize, miR159 was up-regulated by waterlogging and was responsible for silencing two mRNAs encoding GAMYBs, MYB33, and MYB101 homologs (Liu et al., 2012). *MYB33* and *MYB101* together with *MYB65* are controlled by miR159 in *Arabidopsis* to inhibit primary root growth (Xue et al., 2017). Hence, up-regulation of miR159 in maize roots under waterlogging may be important to clear *GAMYB* mRNAs and arrest primary root growth. Also, down-regulation of *GAMYBs* could limit responses related to ABA signaling pathways, in which MYB factors have an active role. On the contrary, miR159 has been found to be down-regulated by flooding in other tissues, such as submerged Lotus seedlings, where degradome and transcriptome analysis confirmed several *GAMYB* targets of miR159 (Jin et al., 2017) that might be involved in the regulation of petiole elongation mediated by GA.

Upon submergence or waterlogging, tolerant plants such as *Alternanthera philoxeroides* respond rapidly to oxygen limitation by initiating adventitious rooting on nodes of the submerged stem (Ayi et al., 2016). Auxin signaling is central to morphological changes in roots through the activity of miRNAs and auxin response factor TFs (ARF; Meng et al., 2010). It has been demonstrated that miR166 is involved in modulating root growth by targeting *HD-ZIP III* transcripts as part of a complex phytohormone response network including auxins (Singh et al., 2017). The over-expression of miR166 resulted in down-regulation of *HD-ZIP III* and increased root growth under normal conditions in *Arabidopsis* (Singh et al., 2014). MiR166 was up-regulated in response to short-term waterlogging in maize roots, where it was demonstrated experimentally to cause down-regulation of its target, the maize *HD-ZIP III* family member *rolled leaf 1* (Zhang et al., 2008). It is possible that during waterlogging, miR166 participates in the modulation of hormone signaling pathways to promote lateral and adventitious rooting through modulation of HD-ZIP III. On the other hand, down-regulation of miR166 was observed in *Arabidopsis* roots under hypoxia stress (Moldovan et al., 2010). Since hypoxia causes the cessation of root growth, which is followed by the rapid development of adventitious roots when normoxia is reestablished, it has been hypothesized that miR166 could play a key role in the pathways that integrate critical levels of hypoxia signals during flooding, like calcium spikes and an increase of ROS, to redirect root growth toward branching or adventitious rooting when normoxia returns.

In addition, in maize roots, miR167 was found to be up-regulated by short-term submergence (Zhang et al., 2008) and short-term waterlogging (Liu et al., 2012). In contrast to short-term stress, miR167 was down-regulated by long-term waterlogging in maize roots, revealing differential regulation during the progression of stress (Zhai et al., 2013). Two miR167 targets were experimentally confirmed in maize roots encoding ARF16 and ARF18 transcription factors (Liu et al., 2012). Based on the previous knowledge that miR167 controls the expression of ARFs to regulate adventitious and lateral root development (Gutierrez et al., 2009), regulation of *ARFs* by miR167 in maize roots could have a balancing effect between suppressing primary root growth and promoting adventitious rooting in response to flooding in a time-scale manner. MiR167 was also up-regulated in the internodes of *Alternanthera* plants (Li et al., 2017) and *Populus* seedlings (Ren et al., 2012) and down-regulated in Lotus seedlings under submersion (Jin et al., 2017), probably to control some of the diverse roles of ARF transcription factors in different tissues (i.e., shoot/petiole elongation) in response to flooding.

In roots of *Arabidopsis* exposed to hypoxia, which is a component of flooding stress, miR390, another miRNA involved in the regulation of auxin response pathways, was up-regulated (Moldovan et al., 2010). It was demonstrated experimentally that miR390 acts by its target mRNA, *TAS3*, from which small trans-acting RNAs are produced (tasiRNA3) that negatively control the expression of ARF TFs. In a study of *Arabidopsis* mutants with altered levels of *TAS3a*, it was found that miR390 and tasiRNA3 were accumulated at the sites for lateral root initiation to block the expression of ARFs and then increase the length of lateral roots. At the same time, ARFs control the auxin-inducible expression of miR390, thus constituting a loop for feedback regulation that maintains ARF expression in a fine-temporal and spatial regulation of the optimal control of root development under oxygen-limited conditions (Marin et al., 2010).

Other miRNAs associated with auxin regulation pathways are miR393 and miR164. MiR393 was down-regulated in response to submergence in Lotus seedlings. As validated by degradome and small RNA sequencing analysis, their targets are mRNAs encoding TIR/F-BOX, which end up being up-regulated by down-regulation of miR393 to promote the auxin response in submerged seedlings (Jin et al., 2017). Conversely, miR393 was up-regulated in waterlogged maize roots (Liu et al., 2012) and submerged *Brachypodium* aerial tissue (Franke et al., 2018). On the other hand, miR164 was up-regulated in maize roots in response to flooding (Liu et al., 2012). It was experimentally demonstrated that in *Arabidopsis* miR164 represses the expression of NAC/NAM domain proteins, and thus provides fine control of lateral root growth promoted by auxins (Guo et al., 2005). This suggests that up-regulation of miR164 in maize roots could have a similar effect on the control of root growth in response to waterlogging.

MiR156 was up-regulated by hypoxia in *Arabidopsis* roots and by submergence in Lotus seedlings and *Brachypodium* aerial tissues (Moldovan et al., 2010; Jin et al., 2017; Franke et al., 2018). MiR156 regulates several mRNAs encoding members of the SQUAMOSA PROMOTER BINDING PROTEIN-LIKEs (SPLs) gene family, which participate in a diversity of roles like control of root growth, transition from the juvenile to the adult phase, and branching and maturation of the shoot (Schwarz et al., 2008). For example, SPL10 is one of the main repressors of root development in *Arabidopsis* (Yu et al., 2015). Under normal conditions, overexpression of miR156 causes the down-regulation of SPL10, thus promoting lateral rooting in *Arabidopsis* (Gao et al., 2018). This suggests that the miR156:SPL10 module might function in regulating the adaptive response of roots in flooding conditions.

During flooding, ethylene functions as a primary signal to induce morphological and metabolic adjustments in plants. APETALA2/Ethylene Responsive Element (AP2/ERF) is a large family of TFs regulated by ethylene that play roles in the control of floral organ identity, in the development of shoot meristem, and in primary and secondary metabolism, among other plant growth and developmental processes (Licausi et al., 2013), and are targeted for posttranscriptional regulation by miR172. Long-term waterlogging in maize roots down-regulated the expression of miR172 and accumulated *AP2/ERF* mRNAs according to degradome analysis (Zhai et al., 2013), suggesting an increase of the ethylene signaling pathways to promote the development of crown roots as an adaptive morphological response.

#### Management of Energy Supply

Flooding creates an environment where an excess of water limits aerobic respiration and photosynthesis, causing a decrease in the energy supply. An alternative for maintenance of the energy supply in such conditions is limiting the futile synthesis of starch and inducing starch breakdown. Submergence in maize roots induces miR399, down-regulating its predicted target, a granule-bound starch synthase (Zhang et al., 2008), suggesting a mechanism to limit the synthesis of starch and avoid wasting energy. In *Populus*, miR399 is also induced in plantlets by submergence and, different to maize, its target mRNA is predicted to encode a Major Facilitator Superfamily protein (MFS) that functions as a transporter of diverse substrates (Ren et al., 2012). In Lotus seedlings, submergence down-regulates several members of the miR399 family, resulting in the accumulation of their predicted target gene encoding a beta-amylase (Jin et al., 2017). Beta-amylase is an enzyme required for starch breakdown active during stress (Wang X. et al., 2017) that could participate in the remobilization of carbohydrates in Lotus to provide energy during flooding.

In maize roots, short-term hypoxia treatment down-regulated the expression of miR159, miR395, and miR474, suggesting the accumulation of their predicted targets encoding enzymes such as vacuolar ATPase, glycoside hydrolase, ATP sulfurylase and malic enzyme (Zhang et al., 2008). These targets are involved in fundamental processes under energy stress, such as ATP production sensing, pH homeostasis, enhancement of sulfur assimilation, carbohydrate breakdown, and pyruvate regulation. However, when hypoxia treatment is continued for more than 24 h and oxygen becomes limiting, miR395 and miR474 are up-regulated to decrease the expression of those enzymes.

#### Control of Flowering

Flowering and the development of floral organs are energy-consuming processes for plants under flooding stress. Thus, delaying flowering and flower development could be seen as an alternative strategy to save energy under submergence stress (Peña-Castro et al., 2011). Despite studies on miRNAs responsive to flooding being mostly performed on roots or seedlings, several miRNAs were described that are involved in controlling flowering during flooding stress. One of them, miR319, is induced in Lotus seedlings to down-regulate the expression of its target *TCP4*, which is critical for petal growth and development (Jin et al., 2017). In this same line of control, miR156 is up-regulated by submergence in *Brachypodium* and Lotus seedlings, and by hypoxia in *Arabidopsis* roots. MiR156 controls the expression of SPLs, which are a rich group of TFs involved in a diverse group of functions in plant tissues. While in roots miR156 could be responsible for the regulation of SPLs involved in root development, in the rest of the plant miR156 may control different SPLs to promote the phase change from vegetative to reproductive growth and other developmental processes like floral organ size and fruit development (Chen et al., 2010).

miR172 also takes part in the response regulating flowering under flooding stress. MiR172 is up-regulated by short-term hypoxia in *Arabidopsis* roots (Moldovan et al., 2010) and by short-term waterlogging in maize roots and Lotus seedlings (Liu et al., 2012; Jin et al., 2017), with regulatory activity over experimentally validated Apetala2 (AP2) AP2/ERF and AGAMOUS TFs, respectively. As SPLs, AP2/ERFs have diverse functions in plant growth depending on the tissue (i.e., in the root), and the severity of the stress. Some targets of miR172 are repressors of flowering, indicating that miR172-mediated down-regulation may promote flowering in plants during the first hours of waterlogging or hypoxia (Zhu and Helliwell, 2011). However, during long-term submergence miR172 is down-regulated, implicating that the target mRNA will be up-regulated, with the consequent repression of flowering. miR5200 is another miRNA involved in flowering that accumulates in *Brachypodium* in response to submergence; it targets FLOWERING LOCUS (FT), a TF with a central role in determining the flowering time, and causes delayed flowering as a strategy for the control of flowering in response to stress (Jeong et al., 2013). In this way, miR5200 contributes to the saving of energy necessary under stress.

#### Oxidative Stress Response

During flooding stress, respiration is impaired, leading to the production of ROS. In order to maintain ROS homeostasis, plants under flooding respond by the activation of oxidation/reduction enzymes, such as cupredoxins. Consistent with this, the most represented biological process predicted from miRNAs responsive to submergence in Lotus seedlings was oxidation–reduction (Jin et al., 2017). Plantacyanin, laccase and superoxide dismutase contain cupredoxin domains and are conserved targets of miR408, miR528, and miR397. In Lotus seedlings, miR408 and miR397 were down-regulated by submergence (Jin et al., 2017). In maize roots under long-term submergence, miR528 was also down-regulated (Zhang et al., 2008). Interestingly, up-regulation was observed for miR408 and miR528 in maize roots undergoing short-term waterlogging (Liu et al., 2012), and for miR397 under short-term hypoxia in *Arabidopsis* roots (Licausi et al., 2011b), reflecting a balance between ROS signaling and toxicity.

In addition to the processes outlined above, other functions could be regulated by miRNAs that await being addressed, such as the potential interplay between responses to flooding and plant–pathogen interaction. In Lotus, two targets encoding putative LRR receptor-like protein kinases, which are recognized for playing a role in response to pathogens, were predicted as targets of miR159 and miR9774. These miRNAs were down-regulated in response to submergence (Jin et al., 2017). On the other hand, miR171 showed up-regulation upon submergence in maize roots and *Brachypodium* (Zhang et al., 2008; Franke et al., 2018), and down-regulation in Lotus seedlings (Jin et al., 2017). MiR171 was predicted to target a *WRKY* TF in maize, which belongs to a large family of TFs that participate in disease resistance networks in plants (Pandey and Somssich, 2009). In *Brachypodium*, miR171 was predicted to target a GRAS TF. Among other functions, GRAS TFs are important in the defense against stress (Mayrose et al., 2006). In Lotus, where miR171 was down-regulated, however, its predicted targets are proteins with unclear or uncharacterized function (Jin et al., 2017).

Fine-temporal regulation of miRNAs is important for the response to flooding stress. Many miRNAs show fluctuating expression profiles during the time-course of hypoxia or submergence treatments (Licausi et al., 2011b; Li et al., 2017). In maize roots, long- and short-term waterlogging accumulated a series of miRNAs with opposite expression profiles depending on the duration of the stress. Most miRNAs up-regulated in the short-term treatment (Liu et al., 2012), were down-regulated under long-term waterlogging (Zhai et al., 2013). Based on the reports of responsive miRNAs to flooding, there is a remarkable trend for down-regulation of miRNAs in mid- and long-term treatments (Zhang et al., 2008; Jin et al., 2017).

When flood-tolerant and flood-sensitive inbred lines of maize were compared, long-term waterlogging down-regulated miRNAs in both lines. However, during the first few hours of waterlogging, in contrast with the sensitive line, the tolerant line up-regulated most of the responsive miRNAs (Liu et al., 2012). This suggests that upon early induction of miRNAs, the repression of functions associated with their target mRNAs, regarded as response signals to hypoxia (e.g., aerenchyma formation, lateral root development and cell detoxification), is a determinant for waterlogging tolerance through the efficient management of energy in oxygen-limited environments. In contrast, in the waterlogging-sensitive line, the down-regulation of miRNAs activated the hypoxia response signals already in the first hours of treatment, thus leaving the plant without the energy and oxygen required to withstand long-term stress.

Understanding the roles of miRNAs in response to flooding can be challenging due to the diversity of mature miRNAs that arise from a single family of miRNAs. Moreover, the mRNAs that are targeted by members of a miRNA family can regulate diverse functions in specific plant tissues. For example, several miRNAs with roles in flowering have been found in the roots of plants during flooding. The functions of these miRNAs and their targets in roots during flooding need to be addressed in order to increase our understanding of the functional diversity of miRNAs. Adding to this complexity, miRNAs function as common elements, connecting the response to different kinds of stress. For example, several miRNAs identified as responsive to flooding stress have also been described in other abiotic stress conditions. In general, all miRNAs related to morphological adaptation and oxidative homeostasis are common to other forms of abiotic stress. Characterizing in detail the expression patterns and function of such miRNAs in the acquisition of tolerance traits in plants represents an opportunity to improve tolerance to flooding in combination with other environmental stress.

The function of miRNAs in flooding stress is estimated by the identity and function of their target mRNAs and consequent downstream effects. In many of the studies mentioned above, the interactions of the miRNA:target modules were confirmed and validated by experimental approaches, while in others, targets were only predicted and need experimental validation. The application of combinatorial approaches, including transcriptome data, degradome, ARGONAUTE immunoprecipitation, and translatome analyses (Jeong et al., 2013; Zhai et al., 2013; Juntawong et al., 2014a; Franke et al., 2018), will be helpful for identifying and consolidating miRNA:target modules and downstream regulation.

In some cases, novel non-conserved miRNAs were identified that are responsive to flooding. These miRNAs are interesting because functional characterization has uncovered new functions potentially explaining group or species-specific traits in plants that have not been described in classic model systems (Ren et al., 2012; Jin et al., 2017; Li et al., 2017). In addition, the identification of miRNAs involved in flooding tolerance and differential strategies for tolerance could be facilitated by analysis of the natural variation using different ecotypes with contrasting tolerance to flooding (Liu et al., 2012) and in flooding-tolerant models like *Sesbania* (Ren et al., 2017), *Oryza* (Nishiuchi et al., 2012), *Rumex*, and *Rorippa* (Voesenek et al., 2014).

Quantitative trait loci associated with morphological traits of interest can also be useful for identifying miRNAs involved in tolerance. For example, in maize, miR166, miR167, and miR319 are co-located with QTLs for tolerance to flooding (Osman et al., 2013). The association of miRNA genes and other genes with QTLs is a powerful strategy for obtaining insight into the molecular basis of quantitative traits and when considering miRNAs as new candidate genes for marker-assisted selection.

Many of the miRNAs responsive to flooding were described and their functions characterized in natural processes like ROS protection and development. The value of some miRNAs for biotechnology was explored, such as of miR156, miR164, miR166, miR172, miR319, and miR159, which have been directed to the improvement of biomass for biofuel production (Chuck et al., 2011; Trumbo et al., 2015). All these miRNAs are regulated by submergence, waterlogging, or hypoxia. However, those miRNAs have not been functionally characterized in detail in the context of tolerance to flooding. This knowledge will open a new area of opportunity for the use of next-generation genomic technologies for the improvement of tolerance to flooding in crop plants mediated by miRNAs.

### Transposon Activation in Submergence/Hypoxia Stress

Much of the current knowledge about plant responses to flooding was obtained through transcriptional studies. It is increasingly recognized that other processes supporting the response can also be important and relevant for agricultural biotechnology (Sanchez, 2013). One of the unexplored pathways in flooding stress is the commonly found, but frequently overlooked, expression of retrotransposons (Mustroph et al., 2010; Rivera-Contreras et al., 2016). Stress has long been known to change the expression patterns of retrotransposons, and the significance of this is hypothesized to have many biological possibilities for acclimatization (Wessler, 1996). With the advent of NGS and accompanying statistical analyses (Xu et al., 2017), a picture has been drawn where transposons may act as transcriptomic enhancers, anchors for novel chromatin methylation patterns, and temporary inducers of expression plasticity in vegetative tissues (Springer et al., 2015; Negi et al., 2016). This knowledge is starting to be compiled as a viable biotechnological strategy to achieve crop improvement (Springer and Schmitz, 2017).

Few reports have explored the role of retrotransposons in flooding stress. Twenty years ago, it was reported that a transposon insertion in the promoter of a tomato 1-aminocyclopropane-1-carboxylate synthase gene (ACC) changed its expression pattern when compared to other members of the ACC family (Shiu et al., 1998). Transposon activity is also characteristic of rice and was detected in several family members (Mustroph et al., 2010).

Transposable elements have been studied in more depth in other kinds of stress. Under drought stress, transposons can also act as miRNA-coding RNAs by acting as promoters of anti-sense transcripts, as observed in maize (Xu et al., 2017). However, given the potential mutagenic action of expressing transposable elements, they are under tight regulation (Li et al., 2010), and natural mechanisms exist that limit their heritability after a stress event, for example, high temperature (Gaubert et al., 2017). The elimination of these controls in plants through different genetic and physiological strategies (including classics like tissue culture), can be a potential biotechnology strategy to induce superior cultivars (Paszkowski, 2015).

However, this elimination not always occurs through traditional expression enhancement, but yet to be characterized mechanisms may be involved that include epigenetic control (Virdi et al., 2015). Recently, an example of a non-canonical role for transposon expression was described in rice roots, showing that their mRNAs can be “miRNA sponges” that act as spurious targets of miRNAs and consequently protect the original target mRNA, and when those mRNAs code for TFs, the effect is amplified (Cho and Paszkowski, 2017). These studies indicate that research of flooding stress response can benefit from testing epigenetic mutation, retrotransposon activity and miRNA bioinformatics.

## Use of Genetic Diversity to Uncover Integrated Mechanisms

Excessive water damages most terrestrial plants. However, genetic diversity in the tolerance to submergence and waterlogging was recognized in many plant species. For example, most rice cultivars die within 7 days under complete submergence (Bailey-Serres et al., 2010). However, a limited number of rice accessions including FR13A can endure complete inundation for up to 14 days (Fukao and Xiong, 2013). QTL analyses identified *SUBMERGENCE-1* (*SUB1*), which positively affects submergence tolerance, on chromosome 9S of FR13A (Xu and Mackill, 1996; Nandi et al., 1997; Sripongpangkul et al., 2000; Toojinda et al., 2003). Introgression of this locus into submergence-intolerant *japonica* and *indica* accessions through marker-assisted backcrossing significantly increased submergence tolerance (Xu et al., 2006; Septiningsih et al., 2009). Map-based cloning and allelic survey revealed that the *SUB1* locus encodes two to three ERF-VII genes, *SUB1A*, *B* and *C*, of which *SUB1A* is the determinant of submergence tolerance (Xu et al., 2006). The major function of *SUB1A* in submergence tolerance is to restrict carbohydrate consumption and amino acid metabolism by the suppression of ethylene production and GA responsiveness and to activate brassinosteroid synthesis, resulting in the restriction of shoot elongation and the avoidance of carbohydrate starvation underwater (Fukao et al., 2006; Fukao and Bailey-Serres, 2008; Barding et al., 2012, 2013; Schmitz et al., 2013; Tamang and Fukao, 2015). Transcriptome analyses indicated that *SUB1A* up-regulates genes involved in ROS detoxification and anaerobic respiration, but down-regulates genes associated with macromolecule biosynthesis (Jung et al., 2010; Mustroph et al., 2010; Locke et al., 2018).

Some rice accessions promote internode elongation in response to flooding, enabling the plants to outgrow gradually rising floodwaters (Fukao and Xiong, 2013). QTL analysis revealed a major locus regulating this response, in which the tandemly repeated ERF-VII genes *SNORKEL1* and *2* are located (Hattori et al., 2009). Consistent with *SUB1A* (Fukao et al., 2006), *SNORKEL1* and *2* are ethylene-inducible (Hattori et al., 2009). It is likely that these deepwater-rice-specific *ERF-VII*s are involved in gibberellin biosynthesis (Ayano et al., 2014). Recent genome-scale gene expression analysis using deepwater and non-deepwater accessions revealed that genes associated with gibberellin biosynthesis and responsiveness, cell wall loosening and extension, and disease resistance are up-regulated in deepwater rice, whereas genes involved in lignin synthesis and oxidative respiration are down-regulated (Minami et al., 2018).

The ability of rice to elongate its seedling shoot under complete submergence is critical for direct seeding. A Myanmar landrace, Khao Hlan On, exhibits strong tolerance of anaerobic germination (Ismail et al., 2009), and QTL analysis identified a major locus responsible, qAG-9-2 (Angaji et al., 2010). This locus contains a gene encoding trehalose-6-phosphate phosphatase, *TPP7*, and a near-isogenic line containing this locus showed increased coleoptile growth underwater (Kretzschmar et al., 2015). Trehalose-6-phosphate functions as an inhibitor of SnRK1 activity in growing tissues (Zhang et al., 2009; Paul et al., 2010; Delatte et al., 2011). The proposed function of TPP7 is to promote the conversion of trehalose-6-phosphate to trehalose, activating the SnRK1-dependent signaling cascade to increase sink strength in growing embryos and coleoptiles (Kretzschmar et al., 2015). In contrast to this *TPP7*-mediated mechanism, *SUB1A* is suggested to restrict the SnRK1-dependent pathway to suppress carbohydrate breakdown in elongating leaves (Locke et al., 2018).

These advances in rice research have not been matched in other crops. Still, studies on QTL identification shed valuable light into what mechanisms, of those known, serve not only as “hardware” of the flooding stress response, but also as natural hubs to increase tolerance. For example, Subtol6, a QTL associated with constitutive higher expression of the HEMOGLOBIN gene in tolerant maize cultivars (Campbell et al., 2015) supports the role of NO signaling, a result that was replicated via RNA-Seq of sensitive and tolerant ecotypes of *Brachypodium* (Rivera-Contreras et al., 2016).

Thorough genetic characterization of the dozens of QTLs associated with populations that have contrasting anatomical adaptations useful for flood stress tolerance, especially in roots, is expected to help to identify novel components (Mustroph, 2018). It is not uncommon to find genes with unknown function in the associated locus (even in model *Arabidopsis*), or multiple and closely located genes promising at the physiological level (e.g., ROS and P signaling; Akman et al., 2017), and awaiting transgenic and introgression analysis (Osman et al., 2013; Zhang et al., 2017).

## Biotechnological Success and Explorations

### *SUB* Locus Introgressions

Identification of the *SUB1* locus and its flanking markers allowed rice breeders to develop rice cultivars that are submergence-tolerant through marker-assisted backcrossing. The submergence-tolerance gene, *SUB1A*, is expressed only during submergence, and its mRNA abundance quickly drops when floodwaters subside (Xu et al., 2006; Fukao et al., 2011). Due to this regulation in time, rice varieties in which the *SUB1* region was introgressed have the same yield, key agronomic traits, and grain quality as the background varieties under regular growth conditions (Singh et al., 2013). On the other hand, *SUB1* rice varieties show greater grain yield after 1–2 weeks of submergence. *SUB1*-introgressed lines were generated by many rice-breeding programs in various countries. Currently, more than four million rice farmers grow SUB1 rice in Asia (Ismail et al., 2013).

### N-Terminal Rule Mutants

Despite the fact that submergence stress is a multicomponent stress, many mutants that were characterized and provided information on the biological mechanisms expressed during submergence/hypoxia have tolerant phenotypes. However, taking aside the well-known case of *SUB1A*, few have been biotechnologically explored in crops. Notably, barley *PRT6* mutants developed by TILLING or RNAi, showed increased tolerance to waterlogging, and maintain pre-stress seed weight (Mendiondo et al., 2016). Further challenging of these mutants proved they are also tolerant to salinity and drought (Vicente et al., 2017), and pathogens (Vicente et al., 2018), although susceptible to *Fusarium*.

### Fruit Conservation and Hypoxia

For a long time, it has been known that fruits respond differentially to ethylene, and this has allowed classifying fruits in two groups depending on their response. Those responsive to ethylene bursts by displaying high respiration rates are named climacteric if the response is strong enough to induce fast ripening, or non-climacteric if the response is milder or slow (Paul and Pandey, 2014). In this way, hypoxic atmospheres have long been used to improve fruit quality, by empirically adjusting the optimal parameters of oxygen and temperature for each genotype, to achieve the desired inhibitory feedback of ethylene production (Lum et al., 2017). Recently, plant signaling and expression studies have reported that CHGs underlie this technology; a network of interacting TFs, including ERF-VIIs, are active and responsible for fermentative metabolism and tannin depletion in persimmon fruit (*Diospyros kaki*), with the practical consequence of decreased astringency (Zhu et al., 2018).

Given that this technology for ripening delay depends on the strength of ethylene feedback inhibition, not only inhibitors of ethylene perception are effective (Lum et al., 2017) but also NO fumigation (Mahajan et al., 2014), a result that is logic now that the role of NO in hypoxia modulation is clearer (see section “Nitric Oxide as a Central Homeostatic Regulator of Hypoxic Stresses”) (Gibbs et al., 2014). However, an excess of hypoxia stress leads to the expression of CHGs and flavor alteration by the accumulation of fermentative products (Cukrov et al., 2016). As mentioned before, the threshold between conservation and fermentation is highly variable in fruits and vegetables (Beaudry, 1999); therefore, a solution to further monitor this balance is the use of metabolomic fingerprinting by combining state-of-the-art analytical methods, informatics, and agronomical knowledge, as recently shown for coffee (Gamboa-Becerra et al., 2017). The use of tissue or developmental stage-specific promoters to drive the timely expression of RNAi constructs against transcripts of ethylene receptors or fermentative enzymes may also render interesting biotechnological prototypes for fruit conservation technology.

### Biomass for Biofuel Production in Flood-Prone Lands

An interesting alternative proposed for employing flood-prone lands in the tropics is the cultivation of sugarcane, which was reported as a flooding-tolerant plant that may even benefit from short flooding periods in the form of increased sugar yields (Glaz and Gilbert, 2006; Ray et al., 2010). The genetic potential of sugarcane was demonstrated in a multi-harvest field-test where family clones of sugarcane, valued for their sugar or biomass yields, were able to retain productivity of both in the face of intermittent flooding (Viator et al., 2012). This ability led to sugarcane being proposed as a component of wastewater treatments in constructed wetlands, where it thrived and removed phosphorus (Mateus et al., 2014).

The use of sugarcane on these types of low-quality soils is being promoted in connection with bioenergy production from the obtained biomass (Viator et al., 2012) since it would not compete in the “food vs. fuel” paradox (Peña-Castro et al., 2017). Research is also being performed to expand the climate range where sugarcane can be cultivated through hybridization with *Miscanthus* (Glowacka et al., 2016), a flood- and chilling-tolerant grass (Mann et al., 2013).

A phenotype that limits the use of other tall grasses as bioenergy crops is lodging (Rueda et al., 2016), i.e., reduction of the stalk’s angle of growth to the soil. Lodging is also a negative consequence of flooding in rice (Jackson and Ram, 2003) and lodging resistance is an agronomic trait that rice breeders intensely focus on (Hirano et al., 2017). A gene coding for gibberellin 2-oxidase, a GA deactivating enzyme, was found to improve lodging resistance (Liu et al., 2018) and members of this gene family are down-regulated in SUB1 rice varieties (Jung et al., 2010). Another example is the allele *SEMIDWARF1* that encodes a gibberellin 20-oxidase, an enzyme of GA biosynthesis, that when active promotes node elongation in deepwater rice in response to flooding, and when silent, is a historic allele that allowed the development of the short high-yield rice of the Green Revolution (Kuroha et al., 2018). Both contrasting examples represent an interesting connection between the fields of bioenergy, submergence tolerance and food security.

In *Jatropha curcas*, a promising biodiesel crop that can produce high oil content in seeds and can be grown on marginal land without competing with other food crops, waterlogging results in significant reduction of growth and biomass yield, implying it is highly sensitive to waterlogging (Gimeno et al., 2012; Verma et al., 2014). Due to its narrow genetic background, the genetic diversity of waterlogging-tolerant *Jatropha* has not yet been reported. However, based on transcriptomic analysis of waterlogged *Jatropha* roots, several candidate genes, including *NR*, *NiR*, and *ERF-VII*s, could be targeted for genetic engineering of waterlogging tolerance (Juntawong et al., 2014b).

### The Potential of Genome Editing for Developing Biotechnological Applications for Submergence/Waterlogging Stress

The advent of editing technologies has opened a new era of discovery in many fields of biotechnology including agricultural research (Doudna and Charpentier, 2014). However, there is only one reported example of gene editing in the field of submergence/waterlogging stress. Yamauchi et al. (2017) edited rice *RESPIRATORY BURST OXIDASE HOMOLOG* truncated genes (*RBOHH*) to demonstrate the crucial role that peroxide produced by this enzyme has on waterlogging signaling and aerenchyma formation. Unfortunately, homozygous plants proved to be sterile and this prevented further phenotyping.

A recent report provides an interesting framework for genome editing for plant biotechnological purposes. Miao et al. (2018) employed Cas9 to create mutants for all *PYRABACTIN RESISTANCE 1* (*PYL*) ABA receptors in rice and explored all combinations in search of genotypes of biotechnological interest. Of all possible combinations, *pyl1/4/6* maximally increased productivity and growth by being released from ABA-related natural growth restrictions but without losing ABA-controlled positive phenotypes, like unwanted seed sprouting, presented by many other mutant combinations. The accumulated knowledge of submergence/flooding stress seems to us to be mature enough to implement this type of powerful gene and genome editing strategies to many of its controlling branches, notably the complex network of TF families.

These promising innovation technologies can produce varieties in which single bases are edited (Chen et al., 2018) up to gene fusions and substitutions (Miki et al., 2018) and even transgene-free genomes (Zhang Y. et al., 2016). The precision and type of induced mutations are less disruptive than many of the mutations empirically conserved during plant domestication history and that prevail in the most valuable traits of major crops of all continents (Meyer and Purugganan, 2013). Regulators must take this into account to avoid disappointing decisions on restraints to the release of gene-edited varieties (Callaway, 2018) and maintain incentives for innovations in research fields where their full potential has not been thoroughly tested, as is the case with submergence/flooding stress.

### Research Structures and Dissemination of Technology

The discovery of the *SUB1* locus and its wide implementation in Asia is a success story in the field of plant biotechnology (Bailey-Serres et al., 2010; Dar et al., 2013; Mickelbart et al., 2015; Dar et al., 2018) and plant molecular biology (Haak et al., 2017). In addition, it should also be a paradigm for research structures that allow the discovery, characterization, and implementation of agricultural phytotechnologies. The research programs of the International Rice Research Institute (IRRI) are solidly based on the diversity of wild and domesticated rice germplasm collected and maintained—for almost a century—in an objective-guided multi-year research plan, and by a committed international scientist nucleus with expertise from agriculture economics to molecular biology (International Rice Research Institute [IRRI], 2017).

Internationally, other CGIAR centers, notably the International Maize and Wheat Improvement Center (CIMMYT), have started to develop similar integral strategies for agricultural research through the agricultural innovation system called Seeds of Discovery–MasAgro (Pixley et al., 2018). This is a strategy aimed at fully employing the maize and wheat germplasm collection using high-throughput genotyping (mostly by NGS) and integrated bioinformatics tools. This framework has produced large-scale genetic data related to flowering time in >4,000 maize landraces (Romero et al., 2017), genetic diversity and its relationship with drought and heat stress of >8,000 wheat accessions in Mexico (Vikram et al., 2016), and the screening of 400 inbred lines of maize and expression analysis of waterlogging-tolerant and -intolerant lines (Du et al., 2017).

The implementation of this specialized genetic characterization as a technology is challenging, especially in countries with a vast range of microclimates such as, e.g., Mexico. Therefore, MasAgro is an innovation platform that connects CIMMYT high-end research and large genetic resources with field-testing, but not in fixed institutionally owned locations, but in a series of hundreds of micro-climate locations where the partner is usually a local company challenging global companies with improved seeds developed for specific locations, or communal associations (Camacho-Villa et al., 2016; Hellin and Camacho, 2017). The result is that genetic information is screened simultaneously in many locations and the most valuable hybrids and landraces are kept and commercialized. These agricultural innovation systems are expected to fill the gap between research and social impact (Westermann et al., 2018). From a scientific standpoint, the flow of genetic material from the lab to the field and back will help develop plant genetic resources valuable for discovering integrated molecular mechanisms that are relevant across environments and flood-related stresses, or to clarify the relative importance, in the context of large genetic-diversity screening, of all those mechanisms herein reviewed and described.

## Conclusion

Scientific understanding of plants’ responses to submergence and waterlogging has dynamically evolved from the pioneering works dealing with fermentative metabolic changes to current state-of-the-art research creating a picture of interconnected perception, transduction and signaling events aimed to support the plant cell in the transit from stress up to the — always expected — recovery phase. These events were demonstrated to involve all possible molecular mechanisms, from mineral signaling to large ribonucleoprotein complexes, in continuous crosstalk, each step adding fitness to the response.

Still, more connections await characterization. In this review we have noted modulation of miRNAs, RNA dynamics and primary energy signaling as some that may benefit from new cutting-edge technologies, developed both inside the field or in parallel areas of plant biology.

The accumulated knowledge prompts us to affirm that the field is sufficiently mature to start moving much of this knowledge to explicit biotechnological tests directed at mitigating the deleterious effects of flooding in the farmer’s economy. The success story of *SUB1*-introgressed lines provides much experience to adapt efforts to other regions of the world commonly affected by this stress.

## Author Contributions

All authors contributed to the research, writing, and review processes for this article.

## Conflict of Interest Statement

The authors declare that the research was conducted in the absence of any commercial or financial relationships that could be construed as a potential conflict of interest.
